# Immune adjuvant effect of a *Toxoplasma gondii* profilin-like protein in autologous whole-tumor-cell vaccination in mice

**DOI:** 10.18632/oncotarget.12316

**Published:** 2016-09-28

**Authors:** Kyoung-Ho Pyo, You-Won Lee, Sun Min Lim, Eun-Hee Shin

**Affiliations:** ^1^ Department of Parasitology and Tropical Medicine, Seoul National University College of Medicine and Institute of Endemic Diseases, Seoul National University Medical Research Center, Seoul, Republic of Korea; ^2^ Division of Medical Oncology, Department of Internal Medicine, CHA Bundang Medical Center, CHA University, Republic of Korea; ^3^ Seoul National University Bundang Hospital, Seongnam, Republic of Korea; ^4^ Current address: JE-UK Laboratory of Molecular Cancer Therapeutics, Yonsei Cancer Institute, Yonsei University College of Medicine, Seoul, Republic of Korea

**Keywords:** Toxoplasma gondii, Toll-like receptor, profilin, antitumor, vaccine

## Abstract

Profilin-like protein in *Toxoplasma gondii* (TgPLP) is a Toll-like receptor (TLR) agonist. In this study, we investigated whether TgPLP has an adjuvant effect on immune function in autologous whole-tumor-cell vaccine (AWV) treatment. Mice vaccinated with AWV together with recombinant TgPLP protein had smaller CT26 tumors and increased survival. TgPLP treatment strongly increased the production of IL-12 through MyD88 signaling and several chemokines, including CCL5, CCL12, and XCL1, in bone marrow-derived macrophages (BMMs). In addition, TgPLP increased the phagocytosis of tumor cells by BMMs and promoted immune cell mobility on a tumor-matrigel scaffold. TgPLP triggered immune responses as demonstrated by increased expression of antigen presenting cell markers (MHC class I and II, B7.1, and B7.2) in BMMs and increased IL-12 and IFN-γ expression in mice. Mice vaccinated with AWV and TgPLP had more immune cells (CD4^+^ and CD8^+^ T cells, natural killer cells, and macrophages) in the spleen and higher total IgG and IgG2a concentrations in the blood than mice vaccinated with AWV alone. These findings suggest that TgPLP is a TLR-based vaccine adjuvant that enhances antitumor immune responses during vaccination with AWV.

## INTRODUCTION

Autologous whole-tumor-cell vaccines (AWVs) are an established treatment for preventing tumor recurrence [1]. Autologous tumor cells are an obvious source of tumor-associated antigens (TAAs), but their use in cancer vaccination is limited by antigen presentation and effector T cell activation [2, 3]. AWVs can be produced from surgically resected tumor tissues, but their therapeutic effect depends upon the co-administration of an immunostimulant [4, 5]. Live cancer cells produce anti-inflammatory cytokines, such as IL-10 or TGF-β, which stimulate immunosuppression by regulatory T cells (Treg) known as suppressor T cells [6, 7]. An alternative strategy to improving the efficacy of AWV is the stimulation of dendritic cells (DCs) or macrophages by TLR signaling, which mediates antigen presentation and increases co-stimulatory surface markers for further T cell function [8].

Toll-like receptors (TLRs) induce IL-12 production via MyD88 signaling and improve vaccine efficacy by enhancing innate immunity and antigen presentation to activate T cells and adaptive immunity [9]. Conserved molecular patterns of microbial pathogens [pathogen-associated molecular patterns (PAMPs)] are TLR ligands [10]. For example, lipopolysaccharide (LPS) is recognized by TLR4 and flagellin is recognized by TLR5 in bacteria. In addition, lipoprotein, lipoteichoic acid, and zymosan are recognized by TLR1, TLR2, and TLR6, respectively [10]. Recently, profilin-like protein was found in the protozoan parasite *Toxoplasma gondii* (TgPLP) and was recognized by TLR11 in mice and TLR5 in humans [11, 12]. TgPLP binds to TLR11 and TLR12 on macrophages and DCs in mice [11, 13], and to TLR5 on human peripheral-blood mononuclear cells (PBMCs) in humans [12]. Profilin contributes to actin polymerization and apicomplexan parasites exhibit an actin-dependent gliding mobility that is essential for migration across biological barriers and invasion of host cells [11]. However, conditional disruption of the *TgPLP* gene in *T. gondii* prevented gliding mobility and TLR 11-dependent IL-12 production by host immune cells [11]. This suggested that TgPLP is an essential component of gliding mobility as like bacterial flagellin and a microbial ligand recognized by the host immune system, both of which are important for *T. gondii* infection [11].

Some TLR agonists can be used as vaccine adjuvants [14]. TLR3 ligands have been experimentally and clinically studied as vaccine adjuvants for HIV, HPV, and cancer [15–18]. Agonists that target TLR7, TLR8, and TLR9 have also been introduced as therapeutic adjuvants for solid tumors and melanomas [19–25]. In particular, bacterial flagellin was used as a TLR5 agonist in cancer therapy [26, 27]. In this study, we investigated whether TgPLP, a TLR11 agonist in mice and a TLR5 agonist in humans, represents a vaccine adjuvant for cancer therapy.

*T. gondii* infection induces cellular immune responses, including IL-12 and IFN-γ production [28]. We have previously shown that *T. gondii* infection and the administration of *T. gondii* lysate antigen (TLA) have an antitumorigenic effect by increasing IL-12 production and decreasing CD31 levels [29]. In addition, euthymic and athymic mice produced IL-12 after TLA treatment. The enhanced innate immune response may have decreased the tumor size by increasing IL-12 production [30]. *T. gondii* infection can also induce tumor immunity, for example, by increasing the number of DCs, macrophages, natural killer (NK) cells, and CD4^+^ and CD8^+^ T cells [31, 32]. *T. gondii* infection decreases tumor growth by Th1 immune responses, which activate cytotoxic T cells [33]. B16 tumor-bearing mice showed decreased tumor growth and increased cellular immune responses after treatment with excretory and secretory *T. gondii* antigens [34]. However, the molecules in *T. gondii* that induce antitumorigenic effects have not been identified. TgPLP is a potential candidate because it is a potent IL-12-inducing protein and a TLR agonist, both of which enhance innate immunity [35]. To investigate the antitumorigenic effects of TgPLP, we produced AWVs from CT26 cancer cells and prepared recombinant TgPLP protein. We investigated the TLR-based antitumorigenic effect of TgPLP *in vivo* (in BALB/c mice) and *ex vivo* (in BMMs). Our findings suggest that TgPLP is a novel cancer-vaccine adjuvant, with general applications in the field of tumor vaccination. Our findings suggest that TgPLP can be a new potential cancer-vaccine adjuvant.

## RESULTS

### Antitumor activity after vaccination with AWV and/or TgPLP in CT26 tumor-bearing BALB/c mice

**Figure 1:**
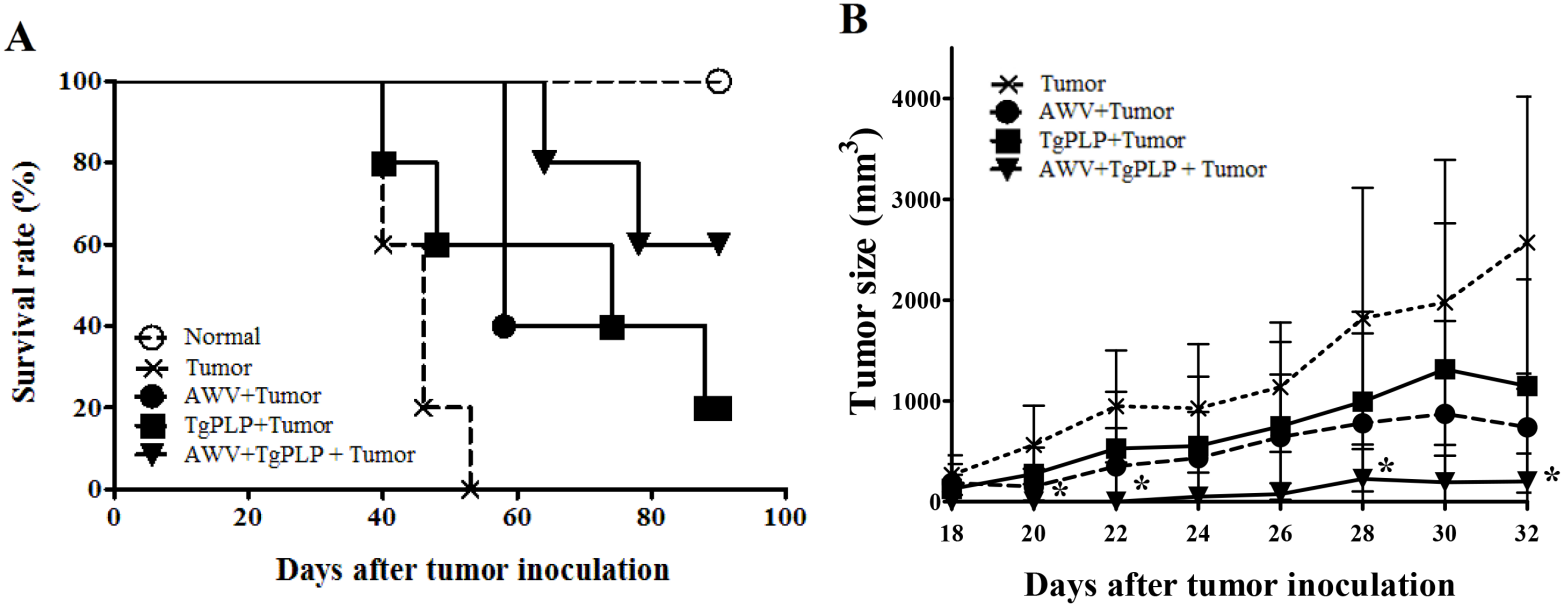
Tumor reduction in CT26-tumor-bearing BALB/c mice vaccinated with AWV and/or TgPLP Survival rate (%) and changes in tumor size (mm^3^) in mice vaccinated with AWV and/or TgPLP were investigated and compared with tumor-bearing mice without vaccination with AWV and/or TgPLP. **A.** Survival rates (%) in each experimental group were designated by the ratio compared with normal mice (all normal mice survived during the experimental period). **B.** Tumor size (mm^3^) in each experimental group between day 18 and 32 after tumor induction. Tumor sizes in mice vaccinated with TgPLP+AWV were significantly decreased compared with tumor-bearing mice without vaccination from day 18 when tumor size was monitored. * indicates statistical significance (p < 0.05).

To confirm the antitumorigenic effects of TgPLP during vaccination with AWV, BALB/c mice were treated with AWV, TgPLP, or AWV+TgPLP. TgPLP protein was produced using bacterial expression system, and confirmed by Western blot on its purity and specificity (Supplementary Figure 1). Mice were vaccinated three times with 1-week intervals before CT26 tumor inoculation (Figure 1). Survival was then monitored in tumor-induced mice for 90 days (Figure 1A). Tumor size was quantified from the 18th day after initiation of tumor formation to the 32nd day (Figure 1B). Tumor mass on the dorsum of mice was significantly decreased in the AWV+TgPLP group compared with the untreated CT26 tumor group at 20, 22, 28, and 32 days after tumor inoculation (*p* < 0.05, Figure 1B). At this time, tumor sizes in other two vaccination group (TgPLP+Tumor and AWV+Tumor) were also decreased; however, the difference was not statistically significant (Figure 1B).

In addition, survival rates were highest in the AWV+TgPLP group with statistical significance (Figure 1A, *P* = 0.0002, log-rank test). CT26-tumor-bearing mice died after 40 days, whereas mice in the AWV+TgPLP group died after 64 days (Figure 1A). All untreated tumor-bearing mice died at day 53, whereas 60% of mice in the AWV+TgPLP group survived until the end of the experiment. Tumor reduction and survival was highest in the AWV+TgPLP group. These findings suggest that TgPLP can be used as a vaccine adjuvant to improve the effect of AWV.

### Stimulation of MyD88-dependent IL-12 production by TgPLP in BMMs

**Figure 2:**
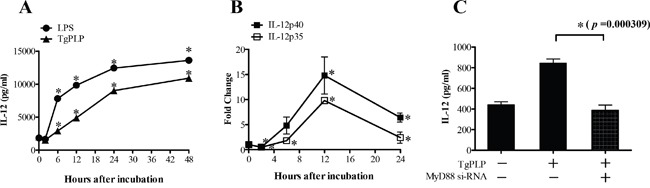
IL-12 production and mRNA expression of the IL-12 subunits (p35 and p40) in BMMs after TgPLP treatment **A.** IL-12 production was significantly increased by the addition of TgPLP (1 μg/ml) (p < 0.05). **B.** The induction of IL-12-p35 and -p40 expression reached peak levels 12 hours after TgPLP treatment as shown by real-time PCR analysis. **C.** IL-12 production induced by TgPLP treatment was dependent upon MyD88 signaling. *MyD88* siRNA in BMMs decreased the production of IL-12. * indicates statistical significance (p < 0.05).

BMMs were used to investigate whether TgPLP increased IL-12 production through TLR-mediated responses *ex vivo*. TgPLP significantly and continuously increased TLR-based IL-12 production by BMMs in culture, similar to LPS treatment as a positive control (*p < 0.05, Figure 2A). *IL-12p35* and *IL-12p40* mRNA levels were increased more than ten times in BMMs at 12 hours after TgPLP treatment (*p < 0.05, Figure 2B). IL-12 production was inhibited by treatment with 1 μg *MyD88*-siRNA (*p* = 0.000309, Figure 2C). Western blot analysis showed that 1 μg *MyD88*-siRNA abrogated the expression of MyD88 protein in BMMs (Supplementary Figure 2). These data suggest that TgPLP induces IL-12 production in BMMs through MyD88 signaling.

### Cytokine and chemokine profiles in BMMs after TgPLP treatment

**Figure 3:**
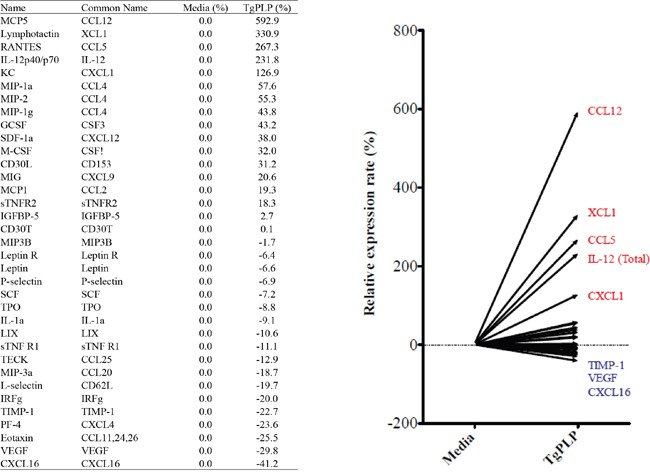
Cytokine and chemokine array of BMMs treated with TgPLP BMMs were incubated with TgPLP (1 μg/ml) for 24 hours. The concentration of cytokines in the culture supernatant was analyzed using a cytokine array kit. Data represent a relative expression rate (%) compared with values in untreated BMMs.

To identify the cytokines and chemokines induced by TgPLP, we analyzed the culture supernatant of TgPLP-treated BMMs by performing a cytokine protein assay using a mouse cytokine array kit. The levels of the following cytokines increased by more than two times after TgPLP treatment: CCL12 (592.9%), XCL1 (330.9%), CCL5 (267.3%), and IL-12p40/p70 (231.8%). In contrast, some cytokines were decreased in the BMM supernatant after TgPLP treatment, for example, eotaxin (−25.5%), VEGF (−29.8%), and CXCL16 (−41.2%) (Figure 3). These results show that TgPLP predominantly induces the production of cell-attractant chemokines and IL-12 by macrophages.

### Increased phagocytosis of tumor cells by BMMs after TgPLP treatment

**Figure 4:**
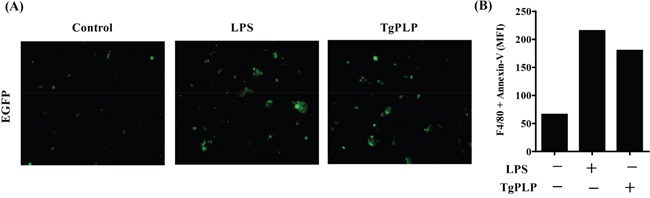
Increased phagocytosis in BMMs after TgPLP treatment **A.** EGFP^+^-CT26-cells were exposed by UV for 10 min, and then used as target cells. The increase in phagocytic ability of BMMs after TgPLP treatment was observed by fluorescence microscopy. Green colored spots represent EGFP^+^-CT26-cell-engulfed BMMs. The green colored spots increased in LPS- and TgPLP-treated BMMs. **B.** Wild-type CT26 cells were exposed by UV for 10 min, and then used as target cells. The increase in phagocytic ability of BMMs in the presence of LPS or TgPLP was observed by FACS analysis. Phycoerythrin (PE)-conjugated antibody against F4/80 and FITC-conjugated Annexin V were used for the observation of double positive signals. The data was represented by median fluorescence index (MFI), and showed the increase in BMMs that CT26 cells had been phagocytosed.

The antitumorigenic activity of the TLR agonist TgPLP may be increased by macrophage activation, for example, during phagocytosis, immune cell infiltration, and further cytotoxic T cell activation. To examine whether TgPLP increases the phagocytosis of tumor cells by BMMs, BMMs were co-cultured with enhanced green fluorescent protein (EGFP)-expressing CT26 tumor cells to visualize the phagocytosis of tumor cells in the presence of LPS or TgPLP (Figure 4). BMMs were not phagocytic in the absence of LPS or TgPLP (Figure 4A, Control). In contrast, GFP fluorescence was detected in BMMs treated with LPS or TgPLP, indicating the phagocytosis of tumor cells (Figure 4A). The increase of GFP fluorescence after TgPLP- or LPS treatment compared to untreated control was calculated by ImageJ software as a relative indicator (%); the result shows that GFP-emitted spots after LPS- and TgPLP treatment were increased by 197% and 163%, respectively (data not shown).

The increased phagocytic activity of BMMs under the treatment of LPS or TgPLP was confirmed by FACS analysis using Annexin V- and F4/80-antibodies (Figure 4B). The double-positive signals of Annexin V (designated for UV-treated apoptotic CT26 cells) and F4/80 (designated for BMMs as pan macrophage marker) means that CT26 cells are phagocytosed into BMMs. The result shows that median fluorescence index (MFI) of the double-positive cells with Annexin V and F4/80 was increased by the treatment of TgPLP as like LPS (Figure 4B).

### Increased BMM mobility and infiltration in a tumor cell-transplanted- and TgPLP-treated matrigel scaffold

**Figure 5:**
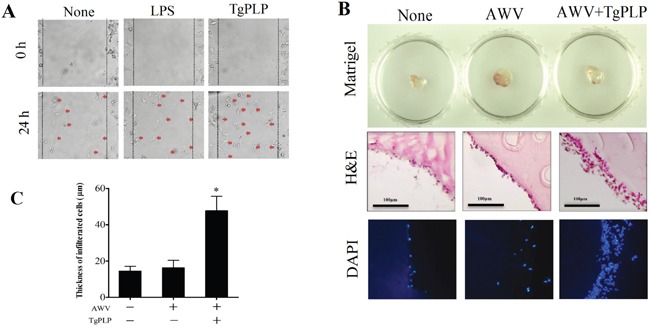
Increased BMM mobility (*in vitro*) and cell infiltration into a matrigel TM scaffold (*in vivo*) after TgPLP treatment **A.** The mobility assay was performed to investigate the change of chemotactic BMM movement after LPS or TgPLP treatment. **B.** The degree of cell infiltration in mice was investigated using matrigel mixed with AWV and/or TgPLP and visualized by H&E staining and DAPI-fluorescence. **C.** The degree of cell infiltration was measured by the infiltrated cell thickness, which was visualized by H&E staining and measured using ImageJ. * indicates statistical significance (p < 0.05).

To examine the effect of TgPLP on immune cell activation for antitumorigenesis, we analyzed BMM mobility *in vitro* in a mobility assay (Figure 5A). In addition, we measured BMM infiltration *in vivo* in a tumor cell-transplanted matrigel scaffold on the dorsum of BALB/c mice (Figure 5B). As revealed by the mobility assay, BMM migration was found to be higher in TgPLP-treated wells, suggesting an increase in the chemotactic movement of TgPLP-treated cells (Figure 5A). Furthermore, recruited cells around the matrigel scaffold on the dorsum of the mice were more abundant in AWV+TgPLP-treated mice, as determined by hematoxylin and eosin (H&E) and DAPI staining (Figure 5B). The abovementioned result was confirmed by the thickness of cell layers surrounding the transplanted matrigels, which were as follows: untreated (14.5 ± 2.6 μm), AWV-treated (16.2 ± 4.1 μm), and AWV+TgPLP-treated matrigels (47.6 ± 8.0 μm) with statistical significance between untreated- and AWV+TgPLP-treated matrigels (*p < 0.05, Figure 5C).

### Increase in antigen presenting cell surface markers after TgPLP treatment

**Figure 6:**
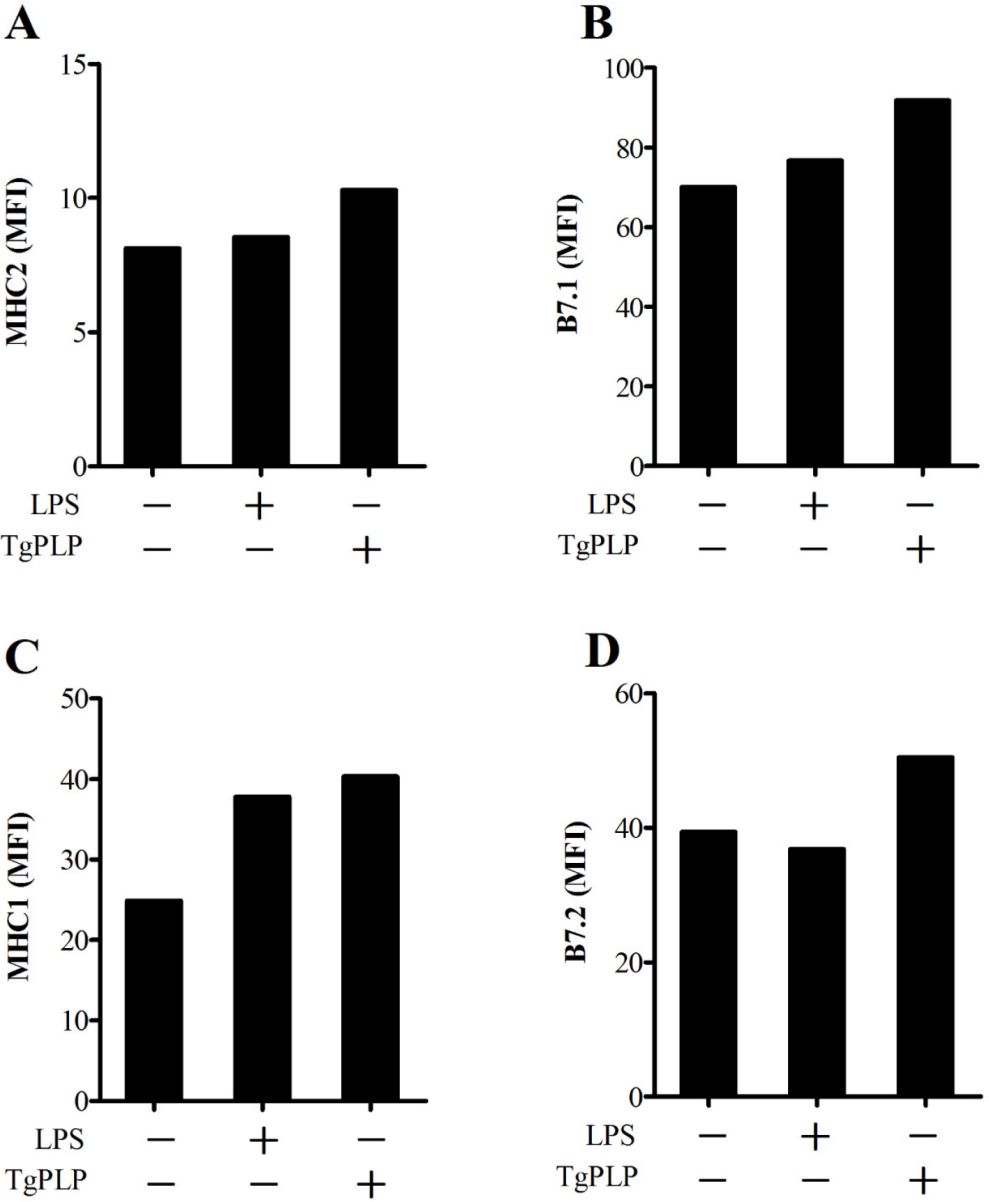
Increase in antigen presenting cell surface markers after TgPLP treatment BMMs were cultured with LPS or TgPLP and the level of cell surface markers, MHC1, MHC2, CD80 and CD86, were measured by FACS analysis. Data are represented as a median fluorescence index (MFI). TgPLP is more effective than LPS at inducing antigen-presentation marker expression.

MHC antigens and co-stimulation receptors are important for the antigen presentation of antigen presenting cells (APCs), such as macrophages and DCs. MHC molecules (I and II) and B7 molecules [CD80 (B7.1) and CD86 (B7.2)] bind to TCR and CD28 on T cells, respectively. These surface proteins have important roles in further adaptive immunity during vaccination with AWV. The effect of TgPLP on the expression of antigen presenting markers was investigated by FACS analysis and shown by the MFI. The expression of MHC molecules (I and II) and B7 molecules [CD80 (B7.1) and CD86 (B7.2)] was higher in TgPLP-treated BMMs compared with LPS-treated BMMs (Figure 6). This suggests that LPS as well as TgPLP can activate macrophages for antigen presentation.

### Cellular and humoral immune characteristics in TgPLP-treated mice

**Figure 7:**
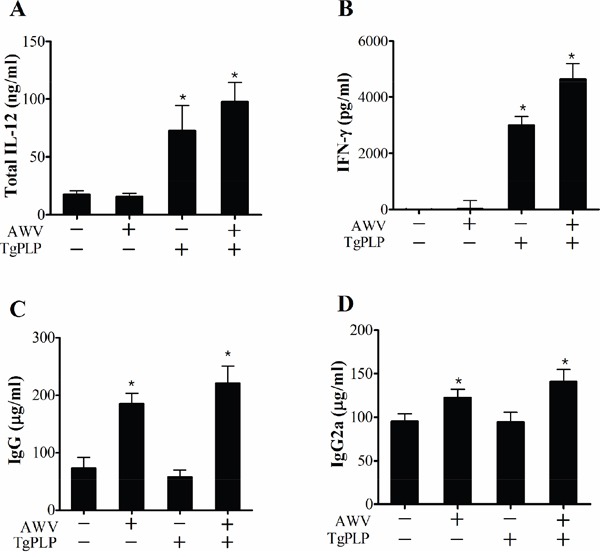
Immune characteristics of BALB/c mice vaccinated with AWV and/or TgPLP After vaccination with AWV and/or TgPLP, mouse sera were collected and cytokines (IL-12 and IFN-γ) and immunoglobulins (total IgG and IgG2a) were measured using ELISA kit. Data represent mean ± SD (n = 5). * indicates statistical significance (p < 0.05).

**Figure 8:**
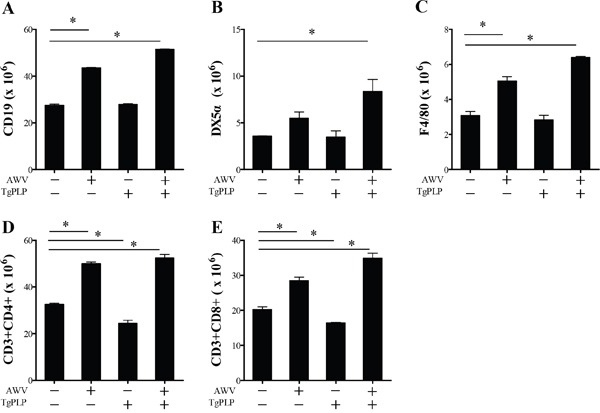
Spleen cell phenotypes of BALB/c mice vaccinated with AWV and/or TgPLP After vaccination, spleens were harvested and cell populations were examined by FACS analysis. Cell numbers were calculated as the ratio of each cell population (CD19^+^, DX5α^+^, F4/80^+^, CD4^+^ or CD8^+^) to total splenocyte number. Data present mean ± SD (n = 5). * indicates statistical significance (p < 0.05).

To investigate the cellular and humoral immune characteristics of mice vaccinated with AWV and/or TgPLP, mice were subcutaneously injected with AWV, TgPLP, or AWV+TgPLP, as described earlier for vaccination with AWV (Figure 7). After vaccination, mice were sacrificed to examine the immune characteristics (cellular and humoral immunity) of the blood and cell phenotypes in the spleen (Figures 7 and 8). Total IL-12 and IFN-γ were significantly increased in TgPLP-treated mice regardless of AWV treatment, and the increase was greater in AWV+TgPLP-treated mice than in TgPLP-treated mice (Figure 7A, 7B). However, IgG and IgG2a levels were significantly increased in AWV-treated mice but not in TgPLP-treated mice (Figure 7C, 7D). This suggests that TgPLP induces cytokines such as IL-12 and IFN- γ production in the blood, whereas AWV can induce humoral immunity as a vaccine effect.

Cell phenotypes in the spleen were examined by flow cytometry (Figure 8). Mice treated with TgPLP alone did not show changes in cell phenotypes (Figure 8). The result was identical to levels in IgG and IgG2a (Figure 7C, 7D). However, AWV treatment increased significantly the number of CD19^+^ B cells, NK cells (DX5a), macrophages (F4/80), CD4^+^ T cells, and CD8^+^ T cells, suggesting the triggering of an immune response. The number of immune cells activated by vaccination with AWV was more increased by TgPLP treatment. This strongly suggests that vaccination with AWV induces antitumor immunity. However, TgPLP is a foreign molecule and cannot induce humoral immunity or trigger cellular immunity by itself. Instead, it enhances antitumor immunity induced by AWV. Taken together, these findings show that TgPLP represents a putative vaccine adjuvant.

## DISCUSSION

Immunotherapies for preventing the progression and relapse of cancer are being improved, including the cancer vaccine, which directly augments anticancer immunity. So far, the development of effective cancer vaccines has had limited success because of characteristic immunosuppression [5]. To develop an effective cancer vaccine, the preparation of TAAs and the development of vaccine adjuvants are necessary to potentiate the immune response [2, 5]. Vaccine adjuvants trigger an early innate immune response against TAAs, which is essential for promoting further adaptive immunity to cancer, such as the activation of cytotoxic CD8^+^ T cells. The TLR response is very important for triggering early immune responses because it is recognized by molecular pattern and not pathogenic specificity. In addition, it easily induces inflammatory cytokines via NF-kb signaling by enhancing antigen presentation [36]. PAMPs trigger TLR signaling in APCs and have important roles in subsequent T cell differentiation and adaptive immunity [37]. For this reason, PAMPs have been used as TLR ligands for cancer immunotherapy [38]. The present study has identified TgPLP derived from *T. gondii* as a novel cancer-vaccine adjuvant.

*T. gondii* infection increases IL-12 production and induces Th1 type host immunity, suggesting the induction of cellular immune response [28]. These host immune characteristics are similar to anticancer immunity, and their antitumorigenic effects have been investigated [29, 30]. We have previously demonstrated that *T. gondii* infection and treatment with TLA decreases the size of S-180 and CT26 tumors in mice [29, 30]. In addition, expression of the angiogenesis marker CD31 (PECAM) was decreased and IL-12 production was enhanced, indicating innate immunity activation [29]. Furthermore, the antitumorigenic effect was observed in euthymic and athymic nude mice, suggesting that this effect was induced by the activation of innate immunity [30, 34]. However, the mechanism for the antitumor effect of *T. gondii* is not completely understood. In this study, we investigated the ability of *T. gondii*-derived TgPLP to induce IL-12 production. TgPLP is a ligand of TLR11 and TLR12 in mouse and TLR5 in human PBMCs [11–13]. TLR signaling in APCs such as macrophages and DCs induces innate and adaptive immune responses, and TLR ligands have been investigated as potential vaccine adjuvants [38]. To investigate this, we examined whether TgPLP treatment increased innate immune responses, including macrophage activation, phagocytosis, cell migration, and antigen presentation. Our findings demonstrated that TgPLP increases IL-12 and IFN-γ production *ex vivo* and *in vivo*. Furthermore, TgPLP increased BMM mobility and immune cell recruitment *ex vivo* and *in vivo*. In addition, TgPLP increased the production of C/CC motif chemokines (CCL12, XCL1, and CCL5), which increase the recruitment and mobility of immune cells and are very important for antitumorigenesis [38]. XCL1 increases chemotactic function and attracts T cells [39]. CCL5 attracts leukocytes and recruits NK cells in tumor tissues [40, 41]. We revealed that the increase in chemokines may enhance BMM mobility and infiltration in TgPLP+AWV-inoculated matrigels. Our results also demonstrated that TgPLP increases the expression of antigen presenting markers such as MHCs (class I and II) and their co-stimulatory receptors (CD80 and CD86), which induce anticancer immunity and phagocytosis. This means that TgPLP-treated cells can induce antigen presentation and adaptive T cell immunity [42]. IL-12 is a major cytokine responsible for polarizing native T cells to Th1 cells [43]. In the present study, we showed that the TLR agonist TgPLP acts as an adjuvant during vaccination with AWV and does not induce adaptive immune responses by itself. Administration of AWV and TgPLP together improved vaccine effectiveness, as shown by enhanced survival and decreased tumor growth.

Taken together, our results have indicated for the first time that TgPLP is a cancer-vaccine adjuvant. TgPLP serves as a PAMP to trigger TLR-MyD88 signaling and IL-12 production. Specific roles of TgPLP include enhancement of cell migration and phagocytosis abilities as well as activation of macrophages. TgPLP does not induce cellular and humoral immunity by itself but augments the effect of AWV when used in combination with it. TgPLP promoted the survival of AWV-vaccinated mice. This suggests that TgPLP represents a novel adjuvant for the treatment of cancer by vaccination with AWV.

## MATERIALS AND METHODS

### Animal ethics

All experiments using BALB/c mice were performed in accordance with the ethical standards of the IACUC at Seoul National University (SNU-140127-2-1). 7-week-old female BALB/c mice were purchased from Orient Bio (Seongnam, Korea) and housed at room temperature with a 12-hour light–dark cycle in SPF condition (Seoul National University Hospital Biomedical Research Institute, Seoul, Korea).

### Preparation of recombinant TgPLP

A 492-bp (165 aa) TgPLP insert was obtained from TGME49_293690 (ToxoDB) without single nucleotide polymorphisms from *T. gondii* strains (type 1: GT1, type 2: ME49, type 3: VEG). The target gene was amplified using a pUC57 (Enzynomics, Daejeon, Korea) vector with a high copy number, inserted into a pQE80L expression vector (N-6-His tagged) (QIAGEN, Hilden, Germany) for protein expression, and then transformed into *E. coli* (BL21) (Enzynomics). After selection using X-gal (Enzynomics) and ampicillin (50 μg/ml) and IPTG induction (0.1 mM), TgPLP recombinant protein was extracted with Ni-NTA beads (QIAGEN). TgPLP bound with Ni-NTA was washed with washing buffer (50 mM NaH_2_PO_4_, 200 mM NaCl, 2 mM imidazole adjusted to pH 8.0 with NaOH). TgPLP protein was eluted with elution buffer (50 mM NaH_2_PO_4_, 300 mM NaCl, 250 mM imidazole, pH 8.0) and desalinated by Amicon Ultra-15 10K (Milipore, Darmstadt, Germany). Then, endotoxins were removed using an endotoxin removal kit (BcMag™, SanDiego, CA). After checking for any remaining endotoxins with a LAL test kit (ToxinSensor™, GenScript, NJ), the final protein solution was aliquoted and stored at −80°C until use. The endotoxin level was <0.01 EU/ml (data not shown). The purity of TgPLP (18 kDa) was confirmed by SDS PAGE and Western blot analysis using an HRP-conjugated anti-His-antibody (Bethyl, TX). A purity of 94% was confirmed using an image analyzer software, ImageJ 1.47V (NIH, MD).

### EGFP-CT26 tumor cells and wild-type CT26 cells

CT26 murine colon carcinoma cells inserted with an EGFP plasmid were provided by Dr. Jung Weon Lee at the Department of Pharmacy, Seoul National University, Seoul, Korea. EGFP-expressing cells were used for *in vitro* phagocytosis assays. Wild-type CT26 cells were purchased from the Korean cell line bank (Seoul, Korea). Cells were cultured in complete DMEM medium (Gibco-Life Technologies, Grand Island, NY) containing 10% FBS (Invitrogen, Carlsbad, CA) and 1% antibiotics–antimycotics (Invitrogen); these cells were then sub-cultured when the cell confluency reached 80% in 100-mm culture dish (SPL, Gyeonggi-do, Korea).

### Preparation of AWV and the effect of vaccination with AWV in BALB/c mice

Tumor formation was induced by injecting 1.0 × 10^4^ CT26 cells subcutaneously into the dorsum of mice. To prepare AWV, 1.0 × 10^6^ CT26 cells were placed in a single microcentrifuge tube and attenuated by freezing in LN_2_ and thawing at 37°C; this freeze–thaw process was repeated five times. For vaccination experiments, mice were divided into five groups (n = 5): normal (untreated), positive control (tumor alone), AWV [tumor induction after AWV (1 × 10^6^ cells/50 μl) was injected three times with a 1-week interval)], TgPLP [tumor induction after TgPLP (50 μg/100 μl) was injected three times with a 1-week interval], and AWV+TgPLP [tumor induction after AWV (1 × 10^6^ cells/50 μl) and TgPLP (50 μg/100 μl) were co-injected three times with a 1-week interval]. AWV and/or TgPLP were subcutaneously injected three times into the dorsum of mice with a 1-week interval. On the following day of the last vaccination, CT26 tumor cells were injected, and tumor growth and survival rates were observed. Tumor size was measured between day 18 and day 32 after tumor induction, and calculated using the following formula: length × width × height × 0.5. Measurements were made using calipers.

### BMMs preparation

BMMs were isolated from BALB/c mice (7-week-old females). Briefly, the bone marrow was isolated by flushing with cold complete RPMI medium containing 10% FBS (Invitrogen) and 1% antibiotics (Invitrogen) from the femur and tibia of hind legs. Cells obtained from the bone marrow were cultured in 100-mm dishes (SPL) in complete RPMI medium containing 30% L929-conditioned medium (a source of M-CSF). Cells were sub-cultured every 3 days. After 2 weeks, the purity was measured by FACSCalibur™ (BD bioscience, CA) after immunostaining for the macrophage marker F4/80. The purity of BMMs exceeded 95% (data not shown).

### Gene knockdown using *MyD88* siRNA in BMMs

The siRNA Reagent System (Santa Cruz Biotechnology, CA) was used for the knockdown of *MyD88* in BMMs. Transfection was performed according to the manufacturer's instructions. Briefly, BMMs were incubated with siRNA transfection medium for 4 hours. siRNA and siRNA transfection reagent (both, 1 μg) were applied to BMMs and then incubated for 24 hours. The transfection efficiency was determined by the transfected yield of FITC-conjugated control siRNA and confirmed to be 59.1% (data not shown). In addition, Western blot analysis was performed to confirm the knockdown of MyD88 protein. MyD88 expression was blocked by the treatment with 1 μg siRNA (data not shown). To investigate MyD88-dependent IL-12 expression, BMMs were cultured with MyD88-siRNA and/or TgPLP (100 ng/ml) in six-well culture plates. Supernatants were collected from cultured BMMs after 24 hours and IL-12 production was measured using an ELISA kit (MABTECH, Nacka Strand, Sweden).

### Quantitation of IL-12 subunits (p35 and p40) by real-time PCR

The expression levels of IL-12p35, IL-12p40, and β-Actin were measured by TaqMan real-time PCR. Primer sequences were as follows: mIL-12p35 forward primer: 5′-CCA CCC TTG CCC TCC TAA AC-3′, reverse primer: 5′- GTT TTT CTC TGG CCG TCT TCA-3′, probe: 5′-FAM- ACC TCA GTT TGG CCA GGG TCA TTC CA-TMRA-3′; mIL-12p40 forward primer: 5′-GGA AGC ACG GCA GCA GAA TA-3′, reverse primer: 5′- AAC TTG AGG GAG AAG TAG GAA TGG-3′, probe: 5′-FAM-CAT CAT CAA ACC AGA CCC GCC CAA-TAMRA-3′; β-Actin forward primer: 5′-AGA GGG AAA TCG TGC GTG AC-3′, reverse primer: 5′-CAA TAG TGA TGA CCT GGC CGT-3′, probe: 5′-FAM-CAC TGC CGC ATC CTC TTC CTC CC-TAMRA-3′. For the PCR reaction, total RNA was extracted using an RNA extraction kit (RNease, QIAGEN), and after checking the integrity and purity of total RNA, 200 ng of total RNA was reverse transcribed using reverse transcriptase premix with oligo d(T)15 primer (EBT-1515, ELPIS biotech, Daejeon, Korea). Five microliter of cDNA was used for real-time PCR with primers (5 pmol) and probe (500 pmol). The PCR conditions were as follows: 40 seconds at 94°C, 15 seconds at 60°C for annealing, and 60 seconds at 72°C for elongation. Amplification was performed using the iQ5 real-time detection system (Bio-Rad Laboratories, Hercules, CA). To assess the fold change of gene expression, Ct values of the housekeeping gene, β-actin, were recorded for all cDNA samples. The signals for cytokine mRNA were normalized by calculating the differences (*_Δ_*Ct) of Ct_β-actin_ and Ct_cytokine_. Data analysis was performed using the *_ΔΔ_*Ct calculation.

### Cytokine array for BMMs treated with TgPLP

To investigate the cytokine profile in BMMs, they were cultured with 1 μg/ml of TgPLP for 24 hours in 6-well plates until 90% confluency. The supernatant of the cultured BMMs was collected and stored at −80°C until use. For the cytokine array, samples were analyzed using RayBio® Mouse Antibody Array-3 (RayBiotech Incorporation, Norcross, GA). The assay was performed according to the manufacturer's instructions. Briefly, the array membranes were incubated with blocking buffer for 12 hours at room temperature. After washing, the membranes were incubated with biotin-conjugated antibody for 12 hours at 4°C, and then incubated with HRP-conjugated streptavidin for 12 hours. The membranes were incubated with detection buffer and exposed by LAS 1000 System (Fuji, Japan). Finally, the scanned image was analyzed by the Multi-Gauge System (Fuji, Japan), and the signal intensity was calculated using ImageJ (NIH). After the normalization process (comparison between normal and positive control), data were represented as the relative expression of cytokines and chemokines [30].

### Phagocytosis in BMMs treated with TgPLP

To investigate the phagocytic activity of BMMs, BMMs were cultured with LPS (50 ng/ml) or TgPLP (500 ng/ml) for 24 hours. EGFP-labeled CT26 cells were exposed to UV radiation for 10 min to have apoptotic characteristics for target cells. After incubation, the attached cells were washed with HBSS three times, and observed by GFP fluorescence microscope, JuLi™ (NanoEnteck, Seoul, Korea). In addition, the phagocytic effect of BMMs was also measured by FACS analysis. For phagocytosis assay by FACS, the target cells, which are the wild type of CT26 cells (Korean cell line back, Seoul, Korea), were exposed by UV for 10 min, and then cultured with BMMs as an effector cells for 24 hours with LPS (50 ng/ml) or TgPLP (500 ng/ml). The result was analyzed by flow cytometry after double staining with phycoerythrin (PE)-conjugated antibody against F4/80 (R&D systems, MN, USA) and FITC-conjugated Annexin V (Biolegend). MFI was measured using FACS data analyzer, FlowJo (Tree Star, OR, USA).

### Mobility assay for cell migration

BMMs were cultured in 6-well plates until 90% confluency. For the mobility assay, the inner bottom of the well was scratched by drawing a line with a white tip, and the movement of BMMs into the scratched space was observed for 24 hours using a JuLi™ cell monitoring system (NanoEnteck, Seoul, Korea) under ×200 magnification. The experimental conditions were as follows: untreated, LPS (50 ng/ml), and TgPLP (500 ng/ml). Cells that moved into the scratched space were represented by a red dot.

### Mobility assay of cells infiltrated into an AWV+TgPLP-matrigel scaffold

Matrigel (400 μl) (BD Bioscience, Bedford, MA) was mixed on ice with 100 μl Hanks' balanced salt solution (Gibco-Life Technologies, Grand Island, NY) containing AWV alone or AWV+TgPLP (500 ng/ml). Matrigel was subcutaneously injected into the dorsum of BALB/c mice anesthetized by a Zoletil® and Rumpun® mixture. After 24 hours, mice were sacrificed and the surrounding tissues including the matrigel were harvested and fixed with 10% formalin for 24 hours. Then, tissues were embedded in paraffin, sliced into 10 μm sections, and stained by H&E. H&E staining was observed under a microscope. Fluorochrome 4′,6-diamidino-2-pheylindole (DAPI) was also observed using a microscope. The thickness of infiltrated cell layers around scaffold was observed by H&E staining and calculated using ImageJ software.

### Expression of antigen-presentation surface marker in BMMs

Antigen-presentation markers (MHC class I and II) and co-stimulation markers (CD80 and CD86) were measured in BMMs by flow cytometry after treatment with LPS (50 ng/ml) or TgPLP (500 ng/ml) for 24 hours. Antibodies for immunostaining were as follows: APC-conjugated anti-MHC1 (1:100, eBioscience, CA), APC-conjugated anti-MHC2 (1:100, eBioscience), PE-conjugated anti-CD80 (1:100, eBioscience), FITC-conjugated anti-CD86 (1:100, eBioscience). For immunostaining, BMMs were incubated with each antibody for 30 minutes at 4°C, washed with FACS buffer three times, fixed in 4% paraformaldehyde, and analyzed by FACSCalibur flow cytometer (BD science). MFI was measured by a FlowJo FACS analyzer (Tree Star).

### Immune characteristics in the blood and spleen of AWV and TgPLP vaccinated mice

BALB/c mice were divided into four groups (n = 5): control, AWV, TgPLP, or AWV+TgPLP. Mice were subcutaneously injected with AWV and/or TgPLP as mentioned above. After vaccination, blood was collected from the facial vein and IL-12, IFN-γ, total IgG, and IgG2a was measured in the sera using sandwich ELISA. To examine cell phenotypes in the spleen, splenocytes were counted and stained with FACS antibodies, and analyzed using FACSCalibur flow cytometer (BD science).

### ELISA for cellular (IL-12 and IFN-γ) and humoral (total IgG and IgG2a) immune response factors

Sandwich ELISA of IL-12 (MABTECH, Nacka Strand, Sweden) and IFN-γ (BioLegend, CA) was performed according to the manufacturer's instructions. Briefly, each capture antibody was coated on a 96 well ELISA plate (Corning® Costar®, NY), and nonspecific binding was blocked with blocking buffer (0.05% Tween-20, 0.1% BSA in PBS). The plate was washed five times with PBS containing 0.05% Tween-20 (PBS-T). Supernatant (50 μl) of BMMs cultured with TgPLP and/or AWV was applied to the plate. After incubation for 2 hours and washing with PBS-T, 100 μl of C17.8-biotin (0.5 μg/ml)-detection antibody was added. After incubation for 1 hour and washing five times, 100 μl of streptavidin-HRP (1:1000) was added. One-hundred microliters of TMB (eBioscience) as a substrate of HRP was added to each well and the optical density was measured by a micro-plate reader (US/E-MAX, Molecular Devices, CA). To measure IFN-γ expression, 100 μl diluted capture antibody was coated to each well. After washing with washing buffer (50 mM Tris, 0.14 M NaCl, 0.05% Tween 20, pH 8.0), the well was blocked with blocking buffer. Each well was treated with 50 μl serum for 2 hours. After washing, 100 μl detection antibody was added and incubated for 1 hour. Finally, avidin-HRP solution was added, followed by treatment with 100 μl TMB. The optical density was measured by a plate reader (TECAN, Männedorf, Switzerland). To measure the total IgG and IgG2a in TgPLP- or AWV-treated mouse sera, a mouse IgG ELISA kit (E90-131, Bethyl) and a mouse IgG2a ELISA kit (E90-107, Bethyl) were used as described above. The detection antibodies were HRP-conjugated and the color reaction was performed with TMP solution. The optical density was measured at 450 nm.

### Phenotypic analysis of splenocytes

Phenotypic changes in splenocytes were analyzed using flow cytometry [44]. Spleens were gently crushed through a stainless-steel mesh under aseptic conditions to remove tissue debris. Spleen erythrocytes were destroyed by hypotonic shock using red blood cell lysis buffer containing NH_4_Cl. After washing, splenocytes were counted using a Trypan blue exclusion test and assigned to FACS staining. Briefly, 100 μl (1 × 10^6^ cells) of this suspension was stained with rat anti-mouse CD16/32 (Fcγ III/II receptor) monoclonal antibodies (mAb) (eBioscience). Primary antibodies for the phenotype determination of splenocytes were as follows: FITC-conjugated anti-mouse mAb against NK cells (CD49b, clone DX5, eBioscience), PE-conjugated anti-mouse mAb against macrophages (F4/80, clone BM8; eBioscience), Cy5-conjugated anti-mouse mAb against CD8^+^ T cells (CD8a (LY-2), clone 53-6.7; eBioscience), and PE-conjugated anti-mouse mAb against CD4^+^ T cells (CD4 (L3T4), clone GK1.5; eBioscience). Staining was performed according to the manufacturer's protocols. Labeled cells were fixed with 4% paraformaldehyde and fluorescence was quantified using a BD FACSCalibur flow cytometer (BD science). Gates were set to exclude nonviable cells and adjusted to detect specifically stained cells. Data were originally expressed as percentage of splenocytes, and then each cell type was calculated in comparison with the total number of spleen cells in each mouse.

### Statistical analysis

Survival rates were estimated by the Kaplan–Meier method and compared between groups with the log-rank test using GraphPad Prism 5.0a software. Statistical analysis was performed by using 1-way analysis of variance (ANOVA) test, and subsequent post hoc comparisons were done using GraphPad Prism 5.0a software. A *p* value of < 0.05 was regarded as significant.

## SUPPLEMENTARY MATERIALS FIGURES


