# Supplementary material for: Immune adjuvant effect of a Toxoplasma gondii profilin-like protein in autologous whole-tumor-cell vaccination in mice
Source: Oncotarget. 2016 Sep 28;7(45):74107–19. doi: 10.18632/oncotarget.12316 (PMC5342039; doi:10.18632/oncotarget.12316)
Supplement: Supplementary file 1 [file oncotarget-07-74107-s001.pdf]

Immune adjuvant effect of a *Toxoplasma gondii* profilin-like protein in autologous whole-tumor-cell vaccination in mice

SUPPLEMENTAL FIGURES

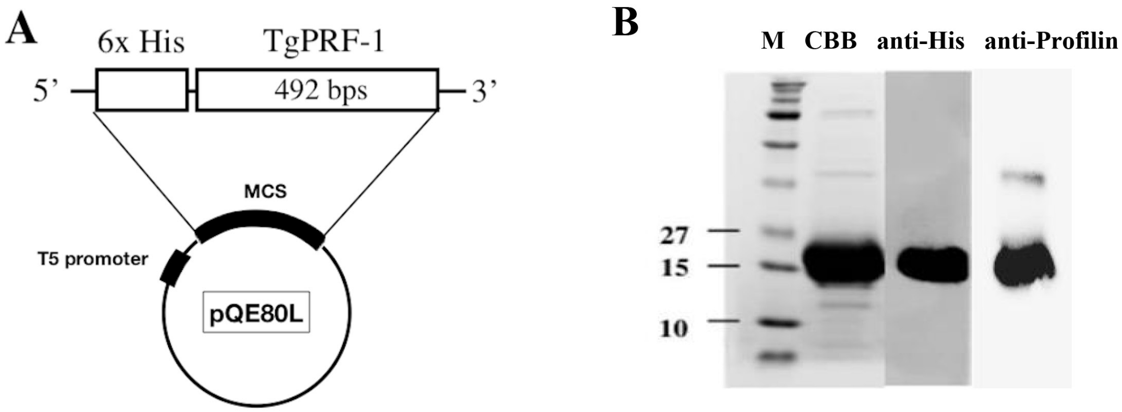

Supplementary Figure S1: Toxoplasma profilin-like protein (TgPLP) was produced using bacterial expression system. The recombinant protein, 492 bps in open reading frame of TgPLP, was expressed as 18-kDa protein bound with N′-His **A.** The purity and specificity of recombinant protein were confirmed by Western blot with anti-His Ab and anti-*Toxoplasma* Profilin Ab **B.**

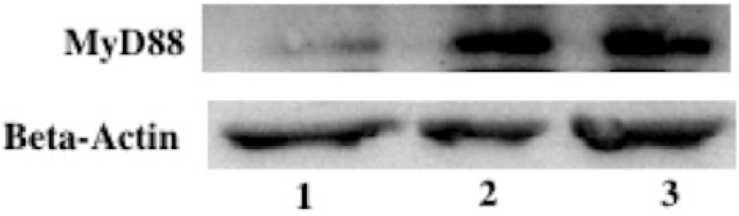

Supplementary Figure S2: Knockdown of MyD88 using siRNA in BMMs. MyD88-siRNA were incubated with BMMs for 24 h. The result of gene knockdown was confirmed by Western blot using anti-MyD88 antibody. Lane 1, 1.0 μg-siRNA; lane 2, 0.5 μg-siRNA; lane 3, 0.25 μg-siRNA. Western blot of beta actin was performed for the confirmation of housekeeping gene expression. When 1 μg of siRNA was added to BMM, MyD88 signaling was blocked.
